# Perception of the Food and Drug Administration Electronic Cigarette Flavor Enforcement Policy on Twitter: Observational Study

**DOI:** 10.2196/25697

**Published:** 2022-03-29

**Authors:** Xinyi Lu, Li Sun, Zidian Xie, Dongmei Li

**Affiliations:** 1 Goergen Institute for Data Science University of Rochester Rochester, NY United States; 2 Department of Clinical & Translational Research University of Rochester Medical Center Rochester, NY United States

**Keywords:** electronic cigarette, FDA flavor enforcement policy, Twitter, Food and Drug Administration, enforcement, policy, e-cigarettes, e-cigarette flavor, tobacco flavors, prohibit, sale

## Abstract

**Background:**

On January 2, 2020, the US Food and Drug Administration (FDA) released the electronic cigarette (e-cigarette) flavor enforcement policy to prohibit the sale of all flavored cartridge–based e-cigarettes, except for menthol and tobacco flavors.

**Objective:**

This research aimed to examine the public perception of this FDA flavor enforcement policy and its impact on the public perception of e-cigarettes on Twitter.

**Methods:**

A total of 2,341,660 e-cigarette–related tweets and 190,490 FDA flavor enforcement policy–related tweets in the United States were collected from Twitter before (between June 13 and August 22, 2019) and after (between January 2 and March 30, 2020) the announcement of the FDA flavor enforcement policy. Sentiment analysis was conducted to detect the changes in the public perceptions of the policy and e-cigarettes on Twitter. Topic modeling was used for finding frequently discussed topics about e-cigarettes.

**Results:**

The proportion of negative sentiment tweets about e-cigarettes significantly increased after the announcement of the FDA flavor enforcement policy compared with before the announcement of the policy. In contrast, the overall sentiment toward the FDA flavor enforcement policy became less negative. The FDA flavor enforcement policy was the most popular topic associated with e-cigarettes after the announcement of the FDA flavor enforcement policy. Twitter users who discussed about e-cigarettes started to talk about other alternative ways of getting e-cigarettes after the FDA flavor enforcement policy.

**Conclusions:**

Twitter users’ perceptions of e-cigarettes became more negative after the announcement of the FDA flavor enforcement policy.

## Introduction

### Background

An electronic cigarette, also known as an e-cigarette or e-cig, is a battery-powered product that typically delivers nicotine in the form of an aerosol [1]. E-cigarette liquid normally contains propylene glycol/vegetable glycerin and flavorings, sometimes contains other additives, such as sweeteners and cannabidiol oil, and frequently contains nicotine [2]. The popularity of e-cigarettes has rapidly increased in recent years, especially among teenagers. According to the 2019 National Youth Tobacco Survey (NYTS), from 2018 to 2019, the proportion of high school current (past 30 days) e-cigarette users increased from 20.8% to 27.5%, and the proportion of middle school current (past 30 days) e-cigarette users increased from 4.9% to 10.5% [3]. Because of the appealing device appearance and various flavor choices, e-cigarettes are more attractive to teenagers and young adults [2].

The e-cigarette flavor choices in the market have rapidly increased in recent years. One study showed that there were more than 460 brands and 7700 unique e-cigarette flavors as of January 2014 [4]. During 2014, the number of e-cigarette brands increased by 10.5 per month, and there were 242 new flavors added each month on average [4]. However, after various federal and state regulations on flavored e-cigarettes, the number of e-cigarette brands and flavors possibly shrank. Among all available e-cigarette flavors, fruit and sweet flavors have been the most popular ones over time [5]. However, the health impact of flavored e-cigarettes is always a major public concern. One study showed that e-cigarette flavorings could lead to endothelial dysfunction, which may increase cardiovascular disease risks [6]. Regardless of the nicotine concentration, e-cigarettes with cinnamon and menthol flavors are more harmful than those with other flavors [6]. Another study showed that both diacetyl and 2,3-pentanedione were associated with changes in gene expression, which impaired both the production and function of the cilia [7]. In addition, our several recent studies found a significant association of e-cigarette use with self-reported wheezing and chronic obstructive pulmonary disease, as well as self-reported difficulty concentrating, remembering, or making decisions in both youth and adults [8-12]. Several studies showed evidence of the effectiveness of e-cigarettes in smoking cessation and reduction with no harm in users up to 2 years, which could potentially benefit current cigarette smokers [13-17]. However, e-cigarettes have no health benefits for youth and adults who have never used any tobacco products before [3,11,18-29].

In order to prevent youth access to flavored e-cigarettes, in November 2018, the Food and Drug Administration (FDA) announced several policies to protect youth, including restricting the sale of flavored e-cigarettes to physical and online stores, with customer age restriction and verification [30]. On January 2, 2020, the FDA announced the flavor enforcement policy that restricted the sale of unauthorized flavored e-cigarette products due to the current epidemic of e-cigarette use among youth [31]. This policy restricted cartridge-based flavored e-cigarette products other than tobacco and menthol flavors, and any e-cigarette product that was targeted to teenagers and young adults. The FDA flavor enforcement policy was implemented on February 6, 2020.

### Objective

To examine the impact of the FDA flavor enforcement policy, we proposed to investigate how the FDA flavor enforcement policy affects the public perception of e-cigarettes and, subsequently, the potential changes in e-cigarette user behavior using Twitter data. Twitter had around 48.35 million active users in the United States in 2019 [32]. Based on the Twitter demographics in 2020, 32% of Twitter users are in the age range of 13 to 17 years, and 38% of Twitter users are in the age range of 18 to 29 years [33]. Similar to the demographics of e-cigarette users, the majority of Twitter users are teenagers and young adults. There are over 5 million youth currently using e-cigarettes. Around 27.5% of high-school students and 10.5% of middle-school students reported using e-cigarettes in 2019 [34]. Twitter data include user information, such as geolocation, which allows us to identify Twitter users from the United States. Twitter has been used in a previous study to examine public reactions to the FDA rule regulating e-cigarettes [35]. Thus, Twitter was chosen as the data source of this research.

In this study, we compared the changes in sentiment toward the FDA flavor enforcement policy and e-cigarettes before and after the FDA flavor enforcement policy. In addition, we tried to examine if there was an intention for potential behavior changes in e-cigarette use with the FDA flavor enforcement policy. The findings of this study provide important insights about the potential effects of the FDA flavor enforcement policy, which could be useful for further policy decision making about the regulation of flavored e-cigarettes to protect public health.

## Methods

### Data Collection From Twitter

E-cigarette–related tweets were downloaded through a Twitter streaming application programming interface (API) using keyword searching based on a list of e-cigarette–related keywords, including “e-cig,” “e-cigs,” “ecig,” “ecigs,” “electroniccigarette,” “ecigarette,” “ecigarettes,” “vape,” “vapers,” “vaping,” “vapes,” “e-liquid,” “ejuice,” “eliquid,” “e-juice,” “vapercon,” “vapeon,” “vapefam,” “vapenation,” and “juul” [5,36,37]. Twitter data were collected during 3 time periods, from June 13, 2019, to August 22, 2019 (before the announcement of the FDA flavor enforcement policy), from January 2, 2020, to February 5, 2020 (between the announcement and the implementation of the FDA flavor enforcement policy), and from February 6, 2020, to March 30, 2020 (after the implementation of the FDA flavor enforcement policy). Tweets from September to December 2019 were not included in this study because during this period many different policies on flavored e-cigarettes were either announced or implemented in different states, such as Michigan, New York, Rhode Island, Oregon, Montana, Washington, New Jersey, and Massachusetts. As a result, a total of 3,874,047 e-cigarette–related tweets were collected after removing retweets.

In order to investigate Twitter users’ perceptions of e-cigarettes within the United States only, another layer of geographic filtering was applied to users’ geolocations. US geolocation keywords that contained both the full names and abbreviations of 50 states in the United States, such as “California,” “Illinois,” and “Florida,” as well as big cities, such as “Los Angeles,” “Chicago,” and “Miami,” were used for filtering. As a result, 2,341,660 e-cigarette–related tweets within the United States were obtained, with 644,686 tweets before the announcement of the FDA flavor enforcement policy, 702,488 tweets between the announcement and implementation of the FDA flavor enforcement policy, and 994,486 tweets after the implementation of the FDA flavor enforcement policy.

Lastly, a third layer of filtering was applied to obtain tweets related to the FDA flavor enforcement policy. The filtering keywords included “FDA ban,” “flavor ban,” “ban,” and any combination of “FDA,” “flavor,” and “ban.” A total of 190,490 FDA flavor enforcement policy–related tweets were collected, with 29,120 tweets before the announcement of the FDA flavor enforcement policy, 89,539 tweets between the announcement and the implementation of the FDA flavor enforcement policy, and 71,831 tweets after the implementation of the FDA flavor enforcement policy. The complete data collection and filtering process is showed in a flowchart in Multimedia Appendix 1.

### Sentiment Analysis

VADER (Valence Aware Dictionary and Sentiment Reasoner) was used as the sentiment analyzer to analyze Twitter users’ thoughts and perceptions of e-cigarettes, and analyze Twitter users’ thoughts and perceptions of the FDA flavor enforcement policy [38]. VADER used a combination of quantitative and qualitative methods by constructing and validating lexical features, which are specifically for microblog-like contexts, as well as combining these lexical features with embodying grammatical and syntactical conventions for expressing and emphasizing sentiment intensity [38]. The overall performance score of VADER sentiment analysis on social media text is the highest compared with the other 8 methods [38]. Every tweet will obtain a sentiment score, ranging from −1.00 to +1.00. According to the suggested threshold for determining positive, neutral, and negative posts, negative posts were defined as tweets with sentiment scores in the range of −1.00 to −0.05, neutral posts were defined as tweets with sentiment scores in the range of −0.05 to +0.05, and positive posts were defined as tweets with sentiment scores in the range of +0.05 to +1.00 [38]. In order to compare the sentiment results between different time periods, the number of tweets in each time period with positive, neural, and negative sentiments was further normalized by the total number of tweets in each time period. In addition, the proportions of positive, neutral, and negative tweets between different time periods were tested by a 2-proportion *Z* test in order to determine the significances of sentiment proportion differences between different periods. The *P* values were calculated with 2-sided testing, and the significant level was set at 5%.

### Topic Modeling

Topic modeling, specifically latent Dirichlet allocation (LDA) modeling, was used to determine the popular topics among e-cigarette–related tweets. LDA is a generative text model for analyzing and clustering words and terms in the given document and generating topics with keywords and their corresponding weights, which indicated the possibility of appearance in the document [39].

According to a research survey published in 2018, the LDA method is one of the most powerful and popular methods used for topic modeling of social network data for knowledge discovery and behavior analysis [40]. A recent paper published in 2020 compared several topic modeling methods for social data analysis and found that the LDA method extracted more meaningful topics than many other topic modeling methods compared [41]. Twitter is one of the most popular social networks that could be used to explore the topics discussed on e-cigarettes and the FDA flavor enforcement policy through LDA modeling.

We applied topic modeling to the e-cigarette tweets in the 3 time periods. First, in order to ensure consistency in the process of the training model, all characters were in lower case, and all words were in the same tense by using the spaCy lemmatization function. In addition, stop words, such as personal pronouns and prepositions, were removed by using Natural Language ToolKit (NLTK) packages. Furthermore, in order to get precise and meaningful results, frequent bigrams (eg, flavor ban) and trigrams (eg, food drug administration) were identified by using the Gensim package, which were considered as a single term rather than separated words during model training. The number of topics was chosen from 3 to 10, and the optimal number of topics was determined by the coherence score of each LDA model result. Lastly, the keywords of the fitted LDA topic model and the percentage distribution of each topic were obtained using the pyLDAvis package.

## Results

### Number of Tweets Related to E-Cigarettes and the FDA Flavor Enforcement Policy

**Figure 1:**
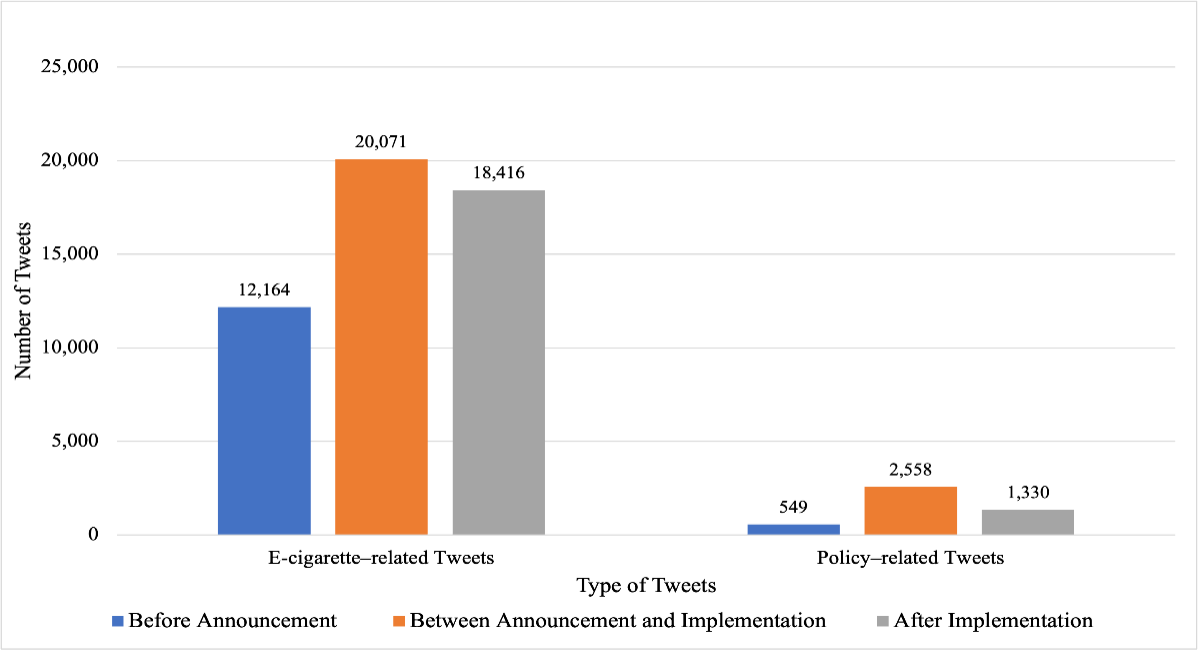
Average number of daily tweets about e-cigarettes and the Food and Drug Administration flavor enforcement policy.

To examine the impact of the FDA flavor enforcement policy on the discussion about e-cigarettes on Twitter, the total amount of tweets was normalized by the number of days before and after the FDA flavor enforcement policy. As shown in Figure 1, the daily average tweets about e-cigarettes and the FDA flavor enforcement policy after the announcement and implementation of the FDA flavor enforcement policy were much higher than that before the announcement of this policy, for example, 12,164 daily e-cigarette–related tweets before the announcement versus 20,071 daily tweets between the announcement and implementation of the FDA flavor enforcement policy. The daily average amount of related tweets after the implementation of the FDA flavor enforcement policy was slightly lower than that between the announcement and implementation of this policy, for example, 18,416 daily tweets versus 20,071 tweets related to e-cigarettes.

### Public Perception of E-Cigarettes and the FDA Flavor Enforcement Policy on Twitter

In order to investigate the perceptions of Twitter users toward e-cigarettes and the FDA flavor enforcement policy, sentiment analysis was conducted, and the proportions of tweets with positive, neutral, and negative sentiments were calculated before and after the FDA flavor enforcement policy. For the better understanding of the sentiment results, Multimedia Appendix 2 included examples for positive, neutral, and negative sentiment tweets from 3 different time periods.

**Figure 2:**
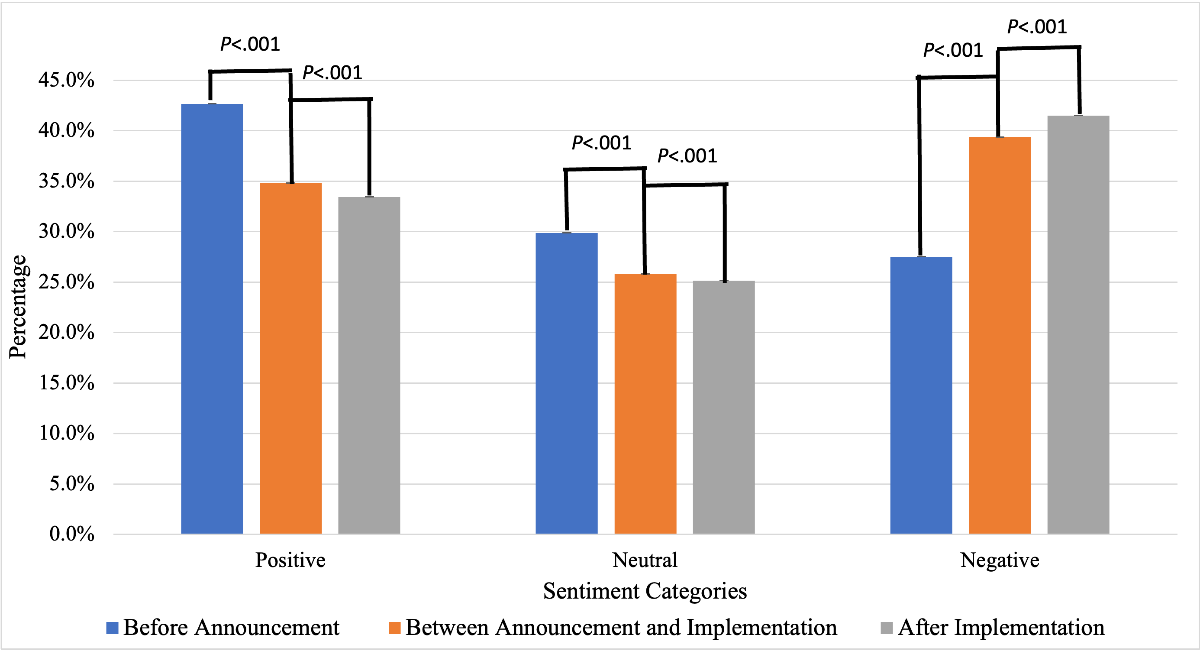
Changes in the public perception of e-cigarettes on Twitter with the announcement and implementation of the Food and Drug Administration flavor enforcement policy.

As shown in Figure 2, the proportion of tweets with positive sentiment toward e-cigarettes decreased significantly (*P*<.001) with the announcement and implementation of the FDA flavor enforcement policy compared to that before the announcement of the FDA flavor enforcement policy, from 42.6% (95% CI 42.5%-42.8%) to 34.8% (95% CI 34.7%-34.9%) and 33.4% (95% CI 33.3%-33.5%). In contrast, the proportion of tweets with negative sentiment toward e-cigarettes significantly increased (*P*<.001) from 27.5% (95% CI 27.4%-27.6%) to 39.4% (95% CI 39.2%-39.5%) and 41.5% (95% CI 41.4%-41.6%). The overall average sentiment score of e-cigarette tweets was positive (0.071) before the announcement of the FDA flavor enforcement policy. In contrast, the overall average sentiment score toward e-cigarettes became negative after the announcement of the FDA flavor enforcement policy, with −0.016 between the announcement and implementation, and −0.024 after the implementation of the FDA flavor enforcement policy.

**Figure 3:**
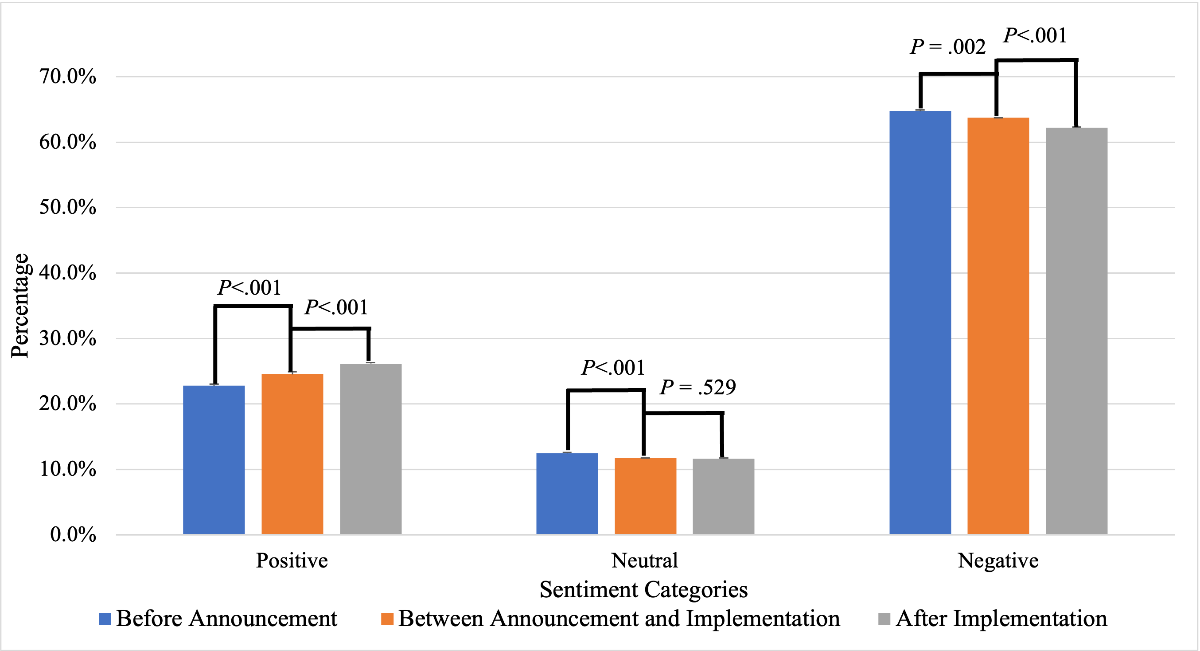
Changes in the public perception of the Food and Drug Administration flavor enforcement policy on Twitter with the announcement and implementation of the policy.

Different from the changes in sentiment toward e-cigarettes, for the FDA flavor enforcement policy–related tweets, the proportion of tweets with positive sentiment increased significantly (*P*<.001) from 22.8% (95% CI 22.3%-23.2%) to 24.5% (95% CI 24.0%-25.0%) and 26.2% (95% CI 25.6%-26.7%), while the proportion of tweets with negative sentiment decreased significantly (*P*=.002 and *P*<.001) with the announcement and implementation of the FDA flavor enforcement policy from 64.8% (95% CI 64.2%-65.3%) to 63.7% (95% CI 63.2%-64.3%) and 62.2% (95% CI 61.7%-62.8%) (Figure 3). For the FDA flavor enforcement policy–related tweets, the overall sentiments were always negative. The average sentiment score was −0.249 before the announcement, −0.246 between the announcement and implementation, and −0.226 after the implementation.

### Topics Related to E-Cigarettes

The LDA topic modeling was applied to e-cigarette–related tweets in order to determine any change in the e-cigarette–related topics discussed by the Twitter users over time. An optimal number of topics was selected by the highest coherence score. Across the 3 time periods, the topics discussing *stop vaping* with the keywords “stop” and “quit” were prevalent (Table 1).

**Table 1:** Top topics related to e-cigarettes before and after the Food and Drug Administration flavor enforcement policy.

Time frame and topic			Tokens, n (%)		Keywords
**Before the announcement (n=644,686)**					
	Stop vaping and smoking to protect health	201,142 (31.20)		vape, vaping, lung, smoke, get, go, people, stop, cancer, health	
	New e-cigarette flavor use among friends	158,593 (24.60)		vape, new, ude, level, link, case, dear_ncan, space_nasking, use, friend	
	Single Juul pod equals a pack of cigarettes	153,435 (23.80)		juul, hit, be, pod, say, still, stare, go, iterally, single	
	Vaping leads to nicotine addiction for those who unlikely smoke	131,516 (20.40)		smoking, cigarette, generation, whole, create, first, start, addiction, unlikely, statistically	
**Between the announcement and implementation (n=702,488)**					
	Ban on flavored tobacco products due to lung disease	231,119 (32.90)		vaping, vape, cigarette, smoking, product, flavor, ban, quit, lung, people	
	Ways to buy vaping products	174,920 (24.90)		vape, would, buy, get, think, shop, go, need, take, look	
	Time to stop vaping and smoking	167,192 (23.80)		smoke, time, vape, juul, stop, early, good, hit, read, drink	
	Epidemic of teenager vaping	129,258 (18.40)		vape, kid, school, new, vapefam, high, top, epidemic, give, vaper	
**After the implementation (n=994,486)**					
	Vaping and smoking have risks to get COVID-19	354,037 (35.60)		vape, vaping, smoking, smoke, want, could, know, risk, covid, people	
	Buy Juul products through shipping	225,748 (22.70)		vape, juul, get, shop, keep, still, flavor, product, sure, ship	
	Intention to stop vaping	210,831 (21.20)		take, vape, stop, late, note, dah, photo, see, guy, friend	
	Vaping and corona virus can cause respiratory disease	202,875 (20.40)		go, lung, vape, virus, people, young, bro, respiratory, disease, affect	

Before the announcement of the FDA flavor enforcement policy, the e-cigarette–related topics included “Stop vaping and smoking to protect health,” “New e-cigarette flavor use among friends,” etc. After the announcement of the FDA flavor enforcement policy, the topic about “Flavor ban” became popular. At the same time, discussions about “Ways to buy vaping products” or “Buy products through shipping” were getting popular. In addition, after the implementation of the FDA flavor enforcement policy (after February 6, 2020), there were increasing discussions about COVID-19 and e-cigarettes with the appearance of “covid” and “virus” keywords in the topics.

## Discussion

### Principal Findings

To ameliorate the high prevalence of e-cigarette use in the United States, especially among teenagers and young adults, the FDA announced and implemented an enforcement policy on flavored e-cigarettes in 2020. In this study, we investigated the changes in perceptions of e-cigarettes and the FDA flavor enforcement policy before and after the announcement and implementation of this policy using Twitter data. In addition, frequent topics discussed together with e-cigarettes by Twitter users were examined.

The proportion of tweets with positive sentiment toward e-cigarettes decreased while the proportion of tweets with negative sentiment increased after the announcement of the FDA flavor enforcement policy compared with before the announcement of the policy. These results suggested that the perceptions of US Twitter users toward e-cigarettes were significantly affected by the announcement and implementation of the FDA flavor enforcement policy. The Twitter users’ perceptions of e-cigarettes in general became more negative with the announcement and implementation of the FDA flavor enforcement policy. Different from e-cigarette–related tweets, tweets about the FDA flavor enforcement policy had opposite trends. The proportion of tweets with positive sentiment increased while the proportion with negative sentiment decreased with the announcement and implementation of the FDA flavor enforcement policy.

### Comparison With Prior Work

Several topics that were frequently discussed with e-cigarettes were common during the 3 time periods, such as health concerns (lung cancer and respiratory disease) and quit vaping, which might be partially due to the occurrence of e-cigarette or vaping product use–associated lung injury in 2019 [42]. After the announcement of the FDA flavor enforcement policy, Twitter users had more discussions about the flavor ban, which was consistent with the increase in the number of tweets about the policy. We showed that the daily average number of policy-related tweets after the announcement of this policy was high. Furthermore, after the announcement of the FDA flavor enforcement policy, topics about alternative ways to get flavored e-cigarettes became one of the most significant themes discussed on Twitter.

To prevent the epidemic of e-cigarette use, the FDA announced several tobacco regulation policies. For example, in April 2014, the FDA released proposed regulation on selling and distributing tobacco products and enhancing the requirement for warning notices on the products [43]. One study conducted an online survey to investigate current smokers’ awareness and perceptions of potential e-cigarette regulation and various policies in the United States [44]. The survey results showed that 83.5% of respondents agreed e-cigarettes should be regulated by the FDA. Although the majority of Twitter users expressed negative emotions in their discussions about the FDA flavor enforcement policy, our study showed that the proportion of Twitter users who expressed positive emotions in their discussions about the FDA flavor enforcement policy slightly increased after the announcement and implementation of the FDA flavor enforcement policy. This might indicate that there were more people supporting the e-cigarette regulation policy and realizing the harm of e-cigarette products after the announcement and implementation of the FDA flavor enforcement policy. Another study was conducted to investigate key conversation trends and patterns over time on Twitter during 2013-2014 [45]. The results showed that “policy and government” was the second most common theme among e-cigarette–related tweets, which indicated that people were willing to discuss and share opinions about e-cigarette policy on Twitter.

With the FDA flavor enforcement policy, the public perception of e-cigarettes became more negative. Furthermore, with the announcement of the FDA flavor enforcement policy, e-cigarette users started to discuss about what they would do, for example, quit vaping or find an alternative. These results suggest that the FDA policy had some significant effects on the use of flavored e-cigarettes, which might potentially change user behavior. A recent survey study examined the effectiveness of the FDA warning label on e-cigarette–related products among college students, and the results showed that the warning label proposed by the FDA was more effective than that created by companies, which reduced more college students’ intentions to use e-cigarettes with the FDA warning notices [46].

During the process of exploring e-cigarette–related conversations on Twitter, we identified topics about the ongoing COVID-19 pandemic and vaping at the beginning of 2020, which was consistent with another social media study on Twitter about COVID-19 and vaping [47]. People had questions on the potential risks of vapers for COVID-19 infection, and whether vaping, linked to lung inflammation, could make individuals more susceptible to COVID-19 infection [47]. However, currently, there is limited evidence about the association between vaping and COVID-19 infection, which needs further investigation.

### Limitations

In this study, Twitter data were used to investigate the changes in the public perception of the FDA flavor enforcement policy. User demographic information, including gender, age, and ethnicity, were not directly available from the Twitter public API, which might limit our further study on the public perceptions of the FDA flavor enforcement policy and e-cigarettes in different demographic groups. Twitter users do not represent the general population, and Twitter users who tagged their geolocation may or may not represent all Twitter users, which might introduce some bias in this study. Moreover, social bots (agents that communicate more or less autonomously on social media) were not identified and removed from the final data, which might bias the results. In this study, we had different numbers of days of Twitter data before and after the FDA flavor enforcement policy to avoid a possible effect of the New York State law on banning all flavored vaper products that was announced on April 3, 2020, and implemented on May 18, 2020, which might cause comparison bias in the results [48]. In addition, people’s actions toward the FDA flavor enforcement policy, such as quit vaping, switch back to smoking, and switch to other flavored e-cigarette products, were not investigated in this study, which will be our future research directions. However, our study did show that e-cigarette users were inclined to find an alternative way to get e-cigarette products.

### Conclusion

Our study showed that the announcement and implementation of the FDA flavor enforcement policy might have influenced Twitter users’ perceptions of e-cigarettes. The findings of this study provide valuable insights into public responses to the FDA flavor enforcement policy, which can be used as an important guideline for future FDA policies on further regulating flavored e-cigarettes.

## Appendix

Multimedia Appendix 1 Flowchart of the data collection and filtering process.

Multimedia Appendix 2 Tweet examples in positive, neutral, and negative sentiment categories.

## Abbreviations

API: application programming interface
FDA: Food and Drug Administration
LDA: latent Dirichlet allocation
VADER: Valence Aware Dictionary and Sentiment Reasoner
