# Supplementary figures and images for: Perception of the Food and Drug Administration Electronic Cigarette Flavor Enforcement Policy on Twitter: Observational Study
Source: JMIR Public Health Surveill. 2022 Mar 29;8(3):e25697. doi: 10.2196/25697 (PMC9006136; doi:10.2196/25697)

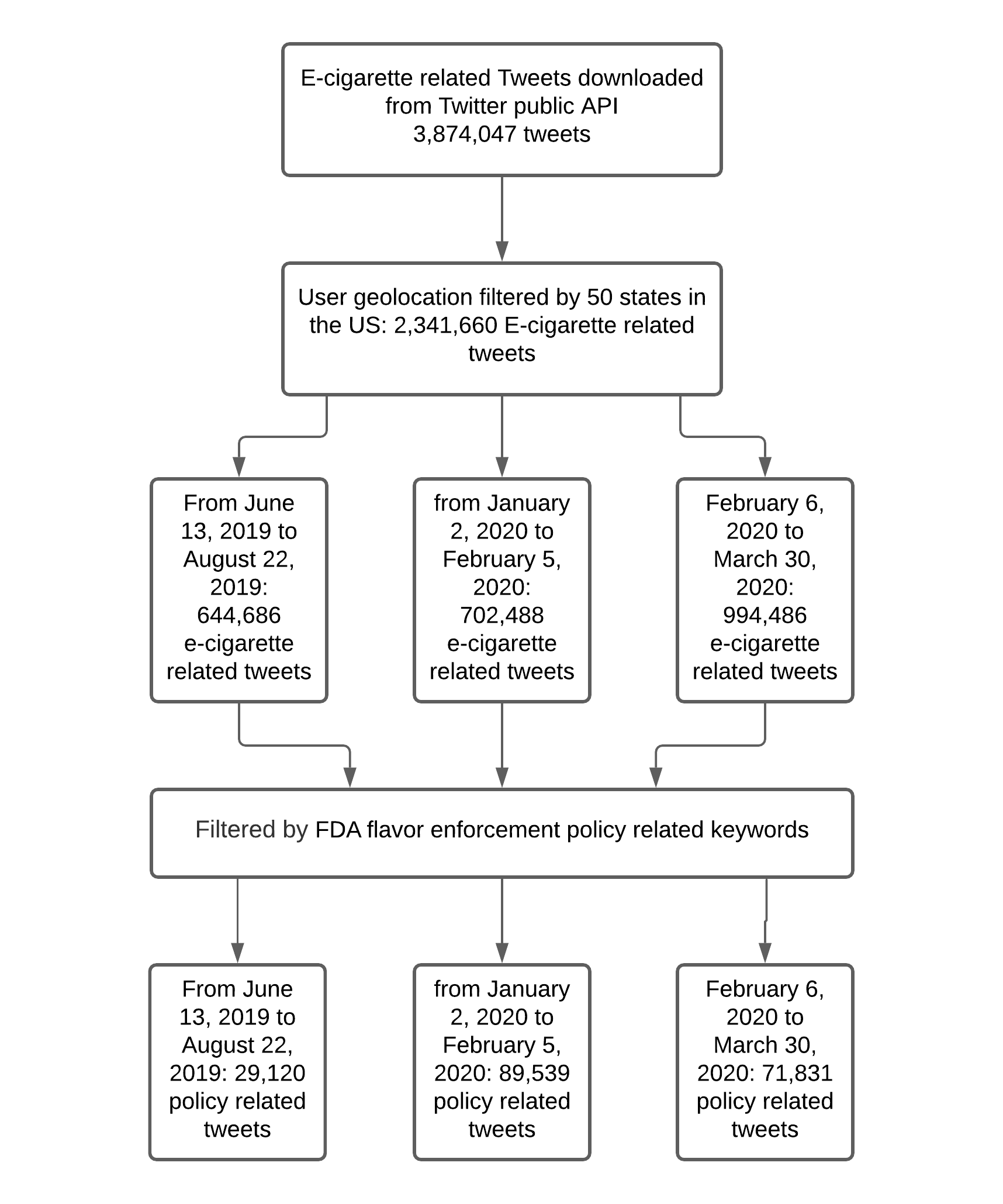

Supplement: Multimedia Appendix 1 [file publichealth_v8i3e25697_app1.png]
