# Supplementary material for: Perception of the Food and Drug Administration Electronic Cigarette Flavor Enforcement Policy on Twitter: Observational Study
Source: JMIR Public Health Surveill. 2022 Mar 29;8(3):e25697. doi: 10.2196/25697 (PMC9006136; doi:10.2196/25697)
Supplement: Multimedia Appendix 2 [file publichealth_v8i3e25697_app2.docx]

**Multimedia Appendix 2.** Tweet examples in positive, neutral, and negative sentiment categories.

|  |  | **Positive Sentiment Examples** | **Neutral Sentiment Examples** | **Negative Sentiment Examples** |
| --- | --- | --- | --- | --- |
| E-cigarettes Related Tweets | Before Announcement | 1. “been juul free for 12 hours and it feels good” 2. “Enjoy vaping everyday. @vape.p.u.s.h.e.r #rdta #vape #vaping #vapecloud” | 1. “Ohm's law and sub ohm vape tanks. Is sub ohming right for you? #subohm #vaping” 2. “My birthday coming up who tryna buy me some juul pods? ” | 1. “Vaping is so stupid. Lung cancer is real and it’s a TERRIBLE way to die, not to mention how painful it is for friends and family to watch that.” 2. “I got the biggest love-hate relationship with my damn juul.” |
|  | Between Announcement and Implementation | 1. “Idk about you, but I LOVE vaping fruity flavors, watermelon is one of my top favorites. If you love fruity juices as much as I do, this flavor is perfect for you! The fusion of the watermelon and strawberries goes so well together, always a favorite.” 2. “Ok everybody! Here is the Spinach Rockstar Vape Pen. This tastes VERY very nice. It’s a very clean taste that packs a punch. The oil is a nice golden beautiful color. What i REALLY love is the three hit modes on the pen. Absolutely perfect in my books.” | 1. “@sabicem Most people switched to vaping in 2017 ish so” 2. “Do you choose your mod or pod based on your style or whatever is the most affordable? #vape #vapefam #smoke” | 1. “My family gets sinus problems from smoking. I was smoking for a while because my stress levels were off the charts. My stress levels decreased by a shit ton so I switched back to gum and vape.” 2. “The CDC estimates that over 20% of high school students use electronic nicotine delivery systems. A group of MCW School of Pharmacy students are coordinating a program at high schools that will raise awareness of the negative health effects of vaping.” |
|  | After Implementation | 1. “I have always been a fan of Aris vapes, but when they came out with this GORGEOUS pink one, I HAD to have it! It's too cute, and works like a gem! I absolutely love the adjustable voltage and how it protects my cartridge! #HappyHabits #HappyHabitsSmoke #HappyHabitsVape” 2. “The juul is a symbol of hope prosperity and eternal friendship. Vmin have turned this d3@dly w3@pon into a product of joy and positivity as they cast their healing light onto the world for others to spread joy and positivity. Amen and god bless” | 1. “thinking abt that time i was cleaning my backpack out during a lecture and found exactly 27 juul pods” 2. “most vape pen makers will transition to synth modules this year i can taste it” | 1. “Because Covid-19 attacks the lungs, those who smoke tobacco or marijuana or who vape may be especially threatened, says Dr. Nora Volkow, director of the National Institute on Drug Abuse” 2. “If the vape industry won't tell the truth about the chemicals, the risks of asthma and cancer and the likelihood of moving onto traditional cigarettes, we will. #vaping #tobacco” |
| FDA Flavor Enforcement Policy Related Tweets | Before Announcement | 1. “Big win for the Vaping industry in New Jersey! In a year that has seen taxes, age restrictions, flavor bans; other issues plague the vaping industry across the US, we now have very good news! New Jersey today scored a victory that we think will help influence other states” 2. “FDA admits #vaping is working to help smokers switch to a safer #nicotine delivery system.” | 1. “San Francisco Bans Vaping for Some Reason. #Trending via @pjmedia_com” 2. “Apparently to keep e-cig away from teenagers the proper measures to take are banning e-cigs for EVERYONE” | 1. “City official 1: We have the highest property crime rate in the nation, the most unaffordable housing, feces everywhere, rampant homelessness and a serious drug abuse problem. What should we do? City official 2: Hmmmm...? How about we ban e-cigarettes!” 2. “Is it possible to BAN SMOKING in any and all public places. I hate having to walk thru smoke or those bloody awful Vape E-cigs which are equally revolting. It’s not ok to blow smoke at others.” |
|  | Between Announcement and Implementation | 1. “@TobaccoFreeKids It is undeniable that smoking cessation should be promoted. It is also a fact that some have quit with FDA approved NRTs. However, there are also a great number of people who have quit with nicotine vaping. #TobaccoHarmReduction must be included for greater success rate.” 2. “#VapingSavesLives saves mine 6 years ago i make one of the best decisions in my life i quit smoking with the help of vaping. As adult and free Individuum i have the right and freedom to choose for myself #vaper #VapeBan #vaping #vapingflavors #Freedom #VapingIsARight"” | 1. “Trump banning vape pods for kids?” 2. “The FDA is rapidly banning vaping and tobacco products for people under 21 so they have healthier soldiers for the draft for WW3 you heard it here first folks.” | 1. “A few people die from vaping. Many more die from assault weapons. People who die from vaping chose to vape. People who die from mass shooting from assault weapons did not choose an assault weapon to kill them. Yet no ban on those. Go figure.” 2. “An estimated 1600 Americans die at the hands of illegal aliens annually. \n\n50 Americans died from vaping this year. The latter was a national crisis that led to a ban.” |
|  | After Implementation | 1. “"How many kids love adult sweetie flavours?? How many adults like kids sweetie flavours?? And why should anyone decide who likes what? Everyone loves flavours!!! Thats the idea you dumb. Nicotine IS NOT the enemy #adultslikeflavours #flavourban #VapeOn #abillionlives” 2. “I never thought I’d be able to stop smoking but honestly the ban on mint juul pods made it super easy I’m so happy I aint got that habit no more.” | 1. “Banning e-cigs from convenience stores could drive smokers to tobacco products, expert says” 2. “Can We Trust The Former FDA Commissioner On Coronavirus After He Caused A Moral Panic About Vaping?” | 1. “1) I don't vape so I don't care if it's banned. 2) why are a lot of the arguments against vaping; for banning about how they can serve as a gateway to smoking cigarettes? If that's the problem, why not, idk, ban cigarettes? 3) no seriously. Why not ban cigs? @truthorange” 2. “Yet they are trying to include a vape ban in a bill for COVID19 testing, hidden at the bottom. I fucking hate it here” |
